# Suppression of Neuroinflammation Attenuates Persistent Cognitive and Neurogenic Deficits in a Rat Model of Cardiopulmonary Bypass

**DOI:** 10.3389/fncel.2022.780880

**Published:** 2022-02-24

**Authors:** Yi Wang, Maro G. Machizawa, Turner Lisle, Cedric L. Williams, Ryon Clarke, Matthew Anzivino, Irving Kron, Kevin S. Lee

**Affiliations:** ^1^Department of Neuroscience, University of Virginia, Charlottesville, VA, United States; ^2^Department of Surgery, University of Pittsburgh Medical Center Pinnacle, Harrisburg, PA, United States; ^3^Center for Brain, Mind and KANSEI Sciences Research, Hiroshima University, Hiroshima, Japan; ^4^Department of Surgery, Vail Health, Vail, CO, United States; ^5^Department of Psychology, University of Virginia, Charlottesville, VA, United States; ^6^Department of Health Sciences, University of Arizona, Tucson, AZ, United States; ^7^Department of Neurosurgery, University of Virginia, Charlottesville, VA, United States; ^8^Center for Brain Immunology and Glia (BIG), University of Virginia, Charlottesville, VA, United States

**Keywords:** neuroinflammation, cardiopulmonary bypass, microglia, neurogenesis, cognitive, minocycline

## Abstract

Post-operative cognitive dysfunction (POCD) can be a serious surgical complication, and patients undergoing cardiac procedures are at particular risk for POCD. This study examined the effect of blocking neuroinflammation on behavioral and neurogenic deficits produced in a rat model of cardiopulmonary bypass (CPB). Minocycline, a drug with established anti-inflammatory activity, or saline was administered daily for 30 days post-CPB. Treatment with minocycline reduced the number of activated microglia/macrophages observed in the dentate gyrus of the hippocampus at 6 months post-CPB, consistent with an anti-inflammatory action in this CPB model. Behavioral testing was conducted at 6 months post-CPB utilizing a win-shift task on an 8-arm radial maze. Minocycline-treated animals performed significantly better than saline-treated animals on this task after CPB. In addition, the CPB-induced reduction in adult neurogenesis was attenuated in the minocycline-treated animals. Together, these findings indicate that suppressing neuroinflammation during the early post-surgical phase can limit long-term deficits in both behavioral and neurogenic outcomes after CPB.

## Introduction

Post-operative cognitive dysfunction (POCD) can complicate surgical outcomes, and is a particular risk for cardiac patients (e.g., Priest et al., 1957; Rasmussen, 2006; Selnes et al., 2006; Fink et al., 2015; Berger et al., 2018). The pathological underpinnings of POCD are likely to be multifactorial, and may include the central spread of microemboli, direct ischemic challenge, and other surgery- and anesthesia-related challenges (e.g., Patel et al., 2012; Berger et al., 2018). Increasing evidence also implicates a key role for neuroinflammation in POCD (Krenk et al., 2010; Hovens et al., 2012; Van Harten et al., 2012; Safavynia and Goldstein, 2019; Jorna et al., 2020), but the manner in which neuroinflammation produces cognitive deficits in POCD remains a matter of discussion. One hypothesis as to how neuroinflammation might contribute to POCD is that activated microglia impair adult neurogenesis in the hippocampus leading to behavioral dysfunction (Hovens et al., 2014, 2016; Wang et al., 2020). Previous evidence has shown that chemically induced neuroinflammation can impair neurogenesis in the adult brain (Ekdahl et al., 2003). And, systemic administration of an anti-neuroinflammatory drug, minocycline, can restore neurogenesis (Ekdahl et al., 2003), raising the possibility that attenuating post-operative neuroinflammation might also mitigate cognitive deficits. Moreover, the potential efficacy of anti-inflammatory medication in POCD was supported by a recent meta-analysis of clinical data (Jorna et al., 2020). Together, these findings suggest that neuroinflammation plays an important role in POCD, and that anti-inflammatory treatment may be an effective remedy.

The prevalence of POCD is highest during the early post-operative period, and most experimental studies have investigated POCD during the first few post-operative weeks (e.g., Hovens et al., 2014). However, POCD can be a chronic condition, sometimes persisting for the lifetime of an individual. A plausible explanation for long-term POCD is that certain aspects of the surgery and/or anesthesia are sufficient to produce focal or distributed cellular damage in the brain, and that neuronal loss underlies the sustained phase of POCD. However, experimental evidence exists supporting the concept that POCD can occur without substantial neuronal loss. For instance, we have shown that cognitive deficits can persist for at least 6 months post-CPB (Wang et al., 2020) in an animal model of CPB that does not produce detectable post-operative neuronal loss (Mackensen et al., 2001; de Lange et al., 2008; Wang et al., 2020). In addition, we have demonstrated that neuroinflammation and reduced adult neurogenesis can persist for at least 6 months in the same experimental model (Wang et al., 2020), consistent with the concept that neuroinflammation is in a position to underlie long-term POCD. The goal of the current study was therefore to evaluate the effects of post-operative suppression of neuroinflammation on persistent neurogenic and behavioral deficits in an experimental model CPB.

## Method

All procedures were approved by the University of Virginia ACUC. Male Sprague-Dawley rats (375–400 g) were randomized to two groups: (1) CPB + Saline or (2) CPB + Minocycline, (*n* = 6/group). The CPB procedure entails a 60-min period of bypass, and was performed as described previously (Grocott et al., 2001; Mackensen et al., 2001; Wang et al., 2020). Beginning 2-h post-surgery, saline or minocycline was administered daily for 30 days according to the protocol of Ekdahl et al. (2003). This treatment protocol has been shown to reduce microglial activation in a model of lipopolysaccharide-induced inflammation. Briefly, this protocol utilizes i.p. injections of minocycline at a dosage of 50 mg/kg, twice daily for the first 2 days and once daily for the next 5 days. During the following 23 days, minocycline was administered at a dosage of 25 mg/kg once daily. Saline injections of the same volume were administered according to the same schedule in the CPB + Saline group. The group identity of all animals was blinded to the investigators performing the behavioral and structural assessments.

At 6 months post-CPB, behavioral testing was performed using a win-shift task on an 8-arm radial maze (Williams et al., 1994). Win-shift is a complex cognitive task in which a multi-stage foraging strategy is used that requires animals to retain and compare information about the location of food over a period of 0.25 to 12 h. Two sequential trials are used separated by a variable inter-trial interval. In the first trial, four food-baited arms are presented in the maze with the other four arms blocked by a clear Plexiglass door. The initial stage of task acquisition is designed such that the second (retention) trial occurs 5 min after the first trial. During the second trial, all of the maze arms are open, but only those maze arms that were not baited in the first trial are now baited. A correct choice in the second trial occurs when an animal visits a baited (i.e., previously unbaited) arm of the maze. An incorrect choice is when an animal visits an unbaited (previously baited) arm. After animals have acquired the task, the duration of the interval between the first and second trials is increased progressively.

Animals were euthanized after behavioral testing for immunohistochemical assessments of activated microglia/macrophages and immature neurons. Rats were deeply anesthetized with isoflurane and transcardially perfused with saline followed by 4% paraformaldehyde in 0.1 M phosphate buffer (PB) pH 7.4. Brains were removed, post-fixed for 2 days, cryoprotected in 30% sucrose, and cryostat sectioned at 30 μm in the coronal plane. Free-floating serial sections at the level of the septal (dorsal) hippocampus were rinsed 3× for 10 min in PB prior to blocking for 60 min in hybridization/blocking buffer containing 5% normal serum of the species in which the secondary antibody was raised, 1% BSA, and 0.3% Triton X-100 in PB. Adjacent serial sections were incubated in the following primary antibodies in hyb/blocking buffer overnight at 4°C at the indicated dilutions: mouse monoclonal anti-CD68 antibody (ED1 clone, 1:250; Abcam ab31630, Cambridge, MA, United States) and a rabbit polyclonal anti-Doublecortin (DCX) antibody (1:500; Abcam, ab18723, Cambridge, MA, United States). Sections were rinsed in PB, exposed to appropriate biotinylated secondary antibodies (all Vector, 1:250) in hyb/blocking buffer for 2 h at room temperature, rinsed, and incubated in avidin-biotin complex (Vector, 1:250) 2 h. Sections were developed using diaminobenzidine with NiSO_4_ intensification, mounted onto Superfrost plus slides (Fisher), dehydrated and cleared through ascending concentrations of ethanol and xylenes, and coverslipped using Permount. Tissue was examined using a Zeiss Axioplan 2 microscope. Stereological cell counting was performed using optical dissector principles by two investigators blinded to the identity of the sections. Eight sections (30 μm thickness) from the septal (dorsal) hippocampus were systematically sampled from each brain. ED1-positive or DCX-positive cells were identified and counted in the granular and infragranular layers of both the internal and external leaves (blades) of the dentate gyrus in each section. An average value for the eight sections was calculated for each animal. These analyses focused on the dentate gyrus in the hippocampus because changes in neurogenesis in the hippocampus are associated with alterations in learning and memory. The analyses did not include assessment of the subventricular zone (SVZ) because a sustained presence of activated microglia/macrophages was not observed in the SVZ after CPB.

## Results

**FIGURE 1:**
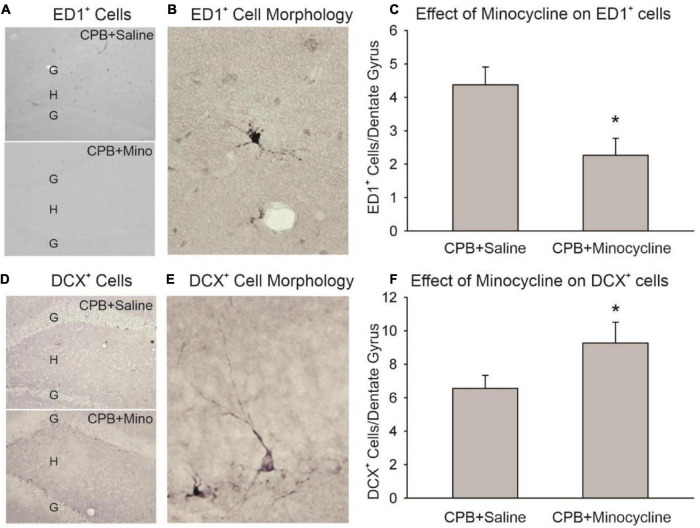
The effect of minocycline on neuroinflammation and neurogenesis. **(A)** Immunohistochemical staining with ED1 in the dentate gyrus. The granule cell layer (G) and hilus (H) of the dentate gyrus are shown in sections from the CPB + Saline and CPB + Minocycline groups. **(B)** An example of the morphology of an ED1-positive (ED1^+^), activated microglia/macrophage in the dentate gyrus. **(C)** Unbiased cell counting was performed for ED1-positive cells at 6 months post-CPB. There were significantly fewer ED1-positive cells in the CPB + Minocycline group as compared to the CPB + Saline group. Values shown are Means and SEMs (*p < 0.05; *t*-test). **(D)** Immunohistochemical staining for DCX in the dentate gyrus in sections from CPB + Saline and CPB + Minocycline groups. **(E)** An example of the morphology of a DCX-positive (DCX^+^), immature neuron in the dentate gyrus. **(F)** Unbiased cell counting was performed for DCX-positive cells at 6 months post-CPB. There were significantly more DCX-positive cells in the CPB + Minocycline group as compared to the CPB + Saline group. Values shown are Means and SEMs (*p < 0.05; *t*-test).

Minocycline treatment during the first month post-CPB reduced the amount of activated microglia/macrophages observed in the hippocampus at 6 months post-CPB, a finding that is consistent with the known anti-inflammatory actions of this compound (Figure 1). The number of ED1-positive cells in the dentate gyrus was significantly lower in the CPB + Minocycline group as compared to the CPB + Saline group. In addition, the number of immature neurons in the dentate gyrus was greater in the treated group (Figure 1). The number of DCX-positive cells was significantly higher in the CPB + Minocycline group as compared to the CPB + Saline group, a finding that is consistent with greater ongoing neurogenesis in the treated group.

**FIGURE 2:**
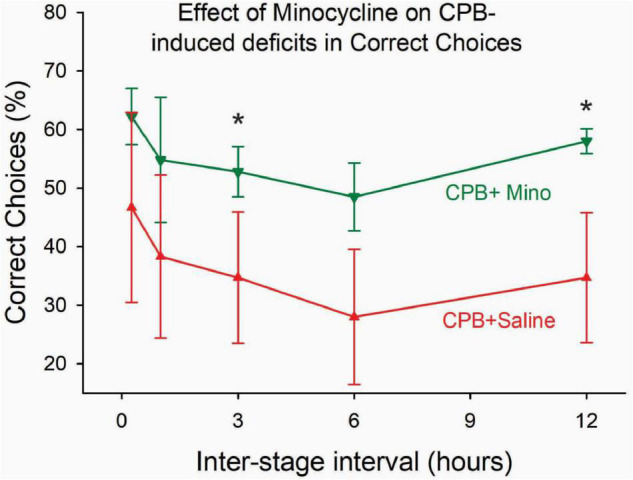
The effect of minocycline on performance on the win-shift task at 6 months post-CPB. Treatment with minocycline improved performance on the task. The number of correct choices on the task was significantly greater in the CPB + Minocycline group at the 3- and 12-h intertrial intervals. Values shown are Means ± SEM (*p < 0.05).

Treatment with minocycline attenuated persistent post-operative cognitive deficits. Performance on the 8-arm, win-shift task at 6 months after surgery was significantly better in the CPB + Minocycline group as compared to the CPB + Saline group (Figure 2).

## Discussion

Surgical and medical advances have greatly improved post-operative outcomes in recent decades. Nevertheless, the risk of POCD remains a concern, particularly in the context of cardiac surgery. Increasing evidence suggests that neuroinflammation plays an important role in POCD (Krenk et al., 2010; Hovens et al., 2012; Van Harten et al., 2012; Safavynia and Goldstein, 2019; Jorna et al., 2020). Although the precise cellular and molecular mediators through which neuroinflammation impacts POCD are still being resolved, one concept is that the activation of microglia suppresses adult neurogenesis, leading to behavioral dysfunction (Hovens et al., 2014, 2016; Wang et al., 2020). Several lines of evidence provide the foundation for this concept. First, chemically induced neuroinflammation results in the activation of microglia and the suppression of neurogenesis (Ekdahl et al., 2003). Second, treatment with an anti-neuroinflammatory drug, minocycline, can attenuate both the activation of microglia and the suppression of neurogenesis (Ekdahl et al., 2003). Third, experimental cardiac surgery can produce neuroinflammation, including the activation of microglia (Hovens et al., 2014; Wang et al., 2020). Fourth, the activation of microglia after cardiac surgery is associated with a reduction in adult neurogenesis (Hovens et al., 2014; Wang et al., 2020). Together these findings raise the possibility that POCD results, at least in part, from a neuroinflammatory response in which activated microglia and/or macrophages reduce adult neurogenesis, leading to cognitive dysfunction.

The present study strengthens this concept by demonstrating that post-CPB treatment with an anti-neuroinflammatory drug: (1) diminishes the activation of microglia, (2) attenuates the suppression of adult neurogenesis, and (3) limits cognitive deficits. Notably, these findings were obtained at 6 months post-surgery using a model of CPB in which substantial neuronal injury does not occur. These findings serve to further implicate neuroinflammation in POCD and, at least in this model, tend to rule out a role for surgically induced neurodegeneration.

It is important to consider the design features of any study with respect to potential complicating factors: (a) The minocycline treatment paradigm utilized in this study [adopted from Ekdahl et al. (2003)] administered drug during the first post-operative month. Consequently, the behavioral testing and cellular assessments were performed 5 months after the last treatment with minocycline. The separation of 5 months between the last drug treatment and testing would rule out a direct drug effect during the testing phase. An important future issue will be to define the optimal timing for drug administration to suppress the CPB-induced neuroinflammatory response. Identification of the time course of early stage neuroinflammation would provide valuable guidance to help refine the timing of treatment. (b) Long-term (i.e., one month) treatment with a drug that possesses antibiotic actions may not be an optimal approach in the post-surgical setting. However, the United States National Library of Medicine states that a person should not take minocycline for longer than 12 weeks, and the present study administered minocycline for just over 4 weeks. In addition, it is important to note that modifications to the structure of minocycline are possible, in order to remove its antibiotic activity while retaining its anti-inflammatory activity. Consequently, future assessments of the efficacy of compounds of this type in the context of long-term POCD could be of considerable value. (c) The use of the ED1 antibody to label CD68-positive cells showed cells with the morphology of activated microglia. However, with this approach it is not possible to distinguish definitively between activated microglia and macrophages that might have been derived from an extra-parenchymal source. Nonetheless, the presence of such cells represents a form of neuroinflammation, irrespective of the source of the cells. (d) Another potential issue is that additional animals receiving sham-surgery were not incorporated into the experimental design. There were two reasons for this aspect of the experimental design. We have previously investigated animals with this treatment and the data are published (Wang et al., 2020). And, in compliance with the intent of the 3-R (Replacement, Reduction, Refinement) principles (Russel and Burch, 1959; Hubrecht and Carter, 2019), that group was not repeated in the present study. This issue is particularly relevant in the current type of study in which blood donor animals are required in addition to the primary experimental animals for the composition of any group. (e) Long-term POCD is a disorder that can last decades. Consequently, it will be important to consider whether the findings described herein will pertain to even longer post-operative survival times. A key advance of the current study is that it evaluated cognitive and neuroinflammatory outcomes at 6 months post-surgery, as compared to time courses of a few weeks utilized in other experimental studies. While the persistence of neuroinflammation and cognitive deficits at 6 months post-surgery is consistent with long-term survival in a rodent, it will be important to investigate these outcomes in another species at longer survival times, in order to further strengthen the findings.

Microglia can play various roles in the physiology and pathophysiology of the central nervous system (CNS), depending on their state of activation. Moreover, beneficial as well as detrimental influences have been defined in the context of CNS disorders (Block et al., 2007; Perry et al., 2010; Gomes-Leal, 2012). The current findings indicate that sustained activation of microglia and/or peripherally derived macrophages play a detrimental role in terms of suppressing adult neurogenesis in the hippocampus after CPB. An important goal for future research will be to identify how an acute peripheral challenge such as CPB can produce sustained central activation of microglia/macrophages.

In summary, the current study provides evidence that post-operative treatment with an anti-neuroinflammatory drug can improve cellular and functional outcomes in a rodent model of CPB. Treatment with minocycline during the first post-operative month reduced microglial activation, limited neurogenesis suppression, and improved behavioral deficits when tested at 6 months post-CPB. These findings therefore provide additional support for a role for neuroinflammation in long-term POCD. In addition, the results are consistent with the concept that activated microglia and suppressed neurogenesis contribute to POCD. Finally, the long-term survival model of CPB utilized herein could prove valuable for assessing the behavioral, neuroinflammatory, and neurogenic effects of therapeutic candidates.

## Data Availability Statement

The raw data supporting the conclusions of this article will be made available by the authors, without undue reservation.

## Ethics Statement

The animal study was reviewed and approved by the University of Virginia ACUC.

## Author Contributions

YW: surgeon, manuscript preparation, and data analysis. MM: behavioral testing. TL: surgeon. CW: behavioral study design. RC and MA: histology. IK: study design. KL: overall study design, data analysis, and manuscript preparation. All authors contributed to the article and approved the submitted version.

## Conflict of Interest

The authors declare that the research was conducted in the absence of any commercial or financial relationships that could be construed as a potential conflict of interest.

## Publisher’s Note

All claims expressed in this article are solely those of the authors and do not necessarily represent those of their affiliated organizations, or those of the publisher, the editors and the reviewers. Any product that may be evaluated in this article, or claim that may be made by its manufacturer, is not guaranteed or endorsed by the publisher.
